# Open access availability of Catalonia research output: Case analysis of the CERCA institution, 2011-2015

**DOI:** 10.1371/journal.pone.0216597

**Published:** 2019-05-07

**Authors:** Anna Rovira, Cristóbal Urbano, Ernest Abadal

**Affiliations:** 1 Department of Librarianship, Information Science and Audiovisual Communication, University of Barcelona, Barcelona, Spain; 2 Research Center on Information, Communication and Culture, University of Barcelona, Barcelona, Spain; Lancaster University, UNITED KINGDOM

## Abstract

The open access availability of publications by Catalonia’s CERCA research centres was analysed to determine the extent to which authors use open access journals, repositories, social networks and other websites to disseminate their research results. A sample of 3,730 journal articles published by authors from CERCA research centres between 2011 and 2015 and available on Web of Science (out of a total output of 44,423) was analysed to identify how many were available in open access, full-text format. The results revealed that 75,8% of the total (2,828 articles) had at least one version available in open access, but just 52% (1,940 articles) had at least one version available in either journals (whether pure or hybrid open access journals or those with embargo periods) or repositories, a finding that highlights the powerful role played by academic social networks in the sharp increase in open access availability. Of the 2,828 articles for which at least one open access version was found, a total of 9,868 copies were located. With respect to versions, the publisher’s final version, i.e. the type formatted for publication by journal publishers, was found in 75,3% of cases. The number of articles published in open access journals (567) was very close to the number of articles published in hybrid journals or journals with embargo periods (624). Only 40,4% of the articles in the sample were located in repositories, being the subject repositories the heaviest used. Fifty percent of the articles (1,881 publications) were posted on academic social networks, the most popular of which were ResearchGate and Academia. According to thematic areas, all six areas (science, life sciences, medical and health sciences, engineering and architecture and humanities) exceeded 70% of articles in open access.

## 1 Introduction

The Budapest Open Access Initiative defined Open Access to scientific communication as:”free availability on the public internet, permitting any users to read, download, copy, distribute, print, search, or link to the full texts of these articles, crawl them for indexing, pass them as data to software, or use them for any other lawful purpose, without financial, legal, or technical barriers other than those inseparable from gaining access to the internet itself. The only constraint on reproduction and distribution, and the only role for copyright in this domain, should be to give authors control over the integrity of their work and the right to be properly acknowledged and cited”. Sixteen years after, interest in open access publication has increased significantly among all scientific communication stakeholders, including researchers, institutions, funding bodies and publishers. As highlighted by Pinfield [1], current questions surrounding this new model focus not so much on whether it will be imposed, but on when and how it will happen.

Moreover, recent European Commission recommendations on access to and preservation of scientific information [2] placed very high demands on member states in terms of obligations and compliance with indicators. Member states have general obligations, but the recommendations also included specific short-term objectives. These referred specifically to open access to science and underpinned the importance of having more accurate and comprehensive information concerning the extent to which researchers, especially those who have secured funding for their projects, publish their work in open access format. For example, they stipulated that all publications resulting from publicly funded projects must be available in open access by 2020 at the latest.

Therefore, a growing number of studies are being conducted to analyse the open access availability of scientific publications both globally and by country or institution type. A brief review of these is provided below.

### 1.1 Background

One of the first such studies was published by Björk [3] and was based on 1,837 articles published in 2008 and indexed in Scopus. The study revealed that 20.4% of all articles analysed were accessible in open access: 8.5% were published in open access journals, while the remaining 11.9% were web-based manuscripts (in repositories or on websites). Given that the study encompassed all fields, the authors were able to determine that the subject area with the weakest presence of open access articles was chemistry, while the most “open” discipline of 2008 was earth science.

Laakso et al. [4] published one of the first longitudinal studies on the evolution of open access journals by analysing a sample from the 1993–2009 period based on journals listed in the Directory of Open Access Journals (DOAJ). They detected a sharp increase in the open access publication of articles and journals during this period. After 2000, the average annual growth rate was 18% for the number of journals and 30% for the number of articles, while there was a 3.5% yearly volume increase in journal publishing in general.

In another longitudinal study, Laakso and Björk [5] measured the volume of scientific articles published in immediate open access and the percentage of open access articles, including those by authors who had paid article processing charges (APCs) to hybrid journals and those published in open access after an embargo period. The authors studied a sample of 787 journals included in DOAJ in 2011 and concluded that journals with APCs accounted for 49% of open access journals and that, of the 19% of the articles published in open access that same year, 12% were available immediately, while the remaining 5% were available 12 months after publication.

Archambault [6] published a comprehensive study on the open access availability of scientific publications during the 2004–2011 period for 22 fields of knowledge in Europe, Brazil, Canada, Japan and the United States. One of the most interesting aspects of the study was the development of a tool to trace 320,000 articles in journals, aggregators and repositories. The results revealed that over 50% of the articles were freely accessible, despite the differences between countries and disciplines.

In 2016, the European Commission [7] presented a report that evaluated the impact of its policy to support open access in seven areas of the Seventh Framework Programme. The findings revealed that 54.2% of the publications resulting from projects in these research areas were available in open access.

In that same year, Laakso and Lindman [8] analysed the relationship between the open access availability of articles from eight information systems journals and the copyright restrictions imposed by their publishers. They concluded that copyright restrictions have very little power to regulate authors’ dissemination of publications and that the use of academic social networks is becoming an increasingly popular practice. This same subject was addressed in a subsequent article by Laakso [9], who focused on publications by researchers at Hanken School of Economics (HSE) and revealed that between 37% and 49% of the articles were available on social networks between 2012 and 2014.

A recent study by Pinowar [10] highlighted the need for large-scale, up-to-date and reproducible studies on open access. In this regard, the authors used three samples, each containing 100,000 articles and the oaDOI service [11]. This service checks, using the DOI, which items are in open access verifying many sources. They conclude that at least 28% of the literature was available in open access. They also found that, when users searched for articles using oaDOI, 47% of the articles were open access. Finally, another study by Science-Metrix [12] included a large-scale analysis of open access publication. They revealed that the level of availability had reached a tipping point whereby at least half of the articles published become available in open access within 12 to 18 months of their publication.

With regard to studies by country, Fathli [13] analysed the state of open access at Swedish universities and the country in general based on 2011 publications in the SwePub database. The results revealed that 10.4% of the articles had been published in open access journals and 9.6% were archived in the repositories of Swedish universities. Elbaek [14] did a follow-up study on open access at Danish universities for the same period and drew similar conclusions. He also wanted to establish a methodology to determine potential open access, i.e. articles that could have a version deposited in repositories because editorial policies allow it.

A study was published by the Research Information Network [15] on the open access situation in the United Kingdom that presented the results of the transition to open access both in the UK and at global level, based on five indicators: the open access options available to authors; the number of articles accessible on open access terms compared to the total number of articles published; the use of open access articles as compared to the total number of articles published; the financial repercussions of open access at universities; and the financial repercussions of open access for learned societies.

Zhang and Watson [16] studied the case of physics projects financed by the Canadian Institutes of Health Research and found that just 22% of the articles were available in open access (13% through green routes and 9% through gold routes). Finally, Bosman and Kramer [17] studied the annual open access levels of Dutch universities based on data from Web of Science and through the oaDOI. They analysed a number of aspects: research areas, languages, countries, institutions, funding bodies and open access types, and observed great diversity across all aspects with regard to open access levels.

With respect to Spain, two studies on the degree of compliance with Article 37 of the Science Act, which stipulates that articles resulting from publicly funded projects be published in open access, presented conflicting data. According to data from Borrego [18], 58.4% of the articles resulting from projects funded by the Spanish government had at least one open access copy available one year after publication, while a study conducted by the Spanish Foundation for Science and Technology [19] concluded that just 9% of the articles resulting from state-funded projects were archived in repositories (and did not appear on other type of websites, as with the other study) between 2011 and 2014. This difference between repositories and social networks was also evident in another study by Borrego [20] that compared the availability of the scientific output of 13 Spanish universities in repositories and on social networks. The author found that just 11.1% of the articles analysed were archived in the researchers’ institutional repositories, while 54.8% were available on ResearchGate. In another vein, Abad-García et al. [21] analysed 762 articles resulting from projects financed by the FIS-2012 of the Carlos III Health Institute with a view to studying the use of OpenAIRE, BASE, RECOLECTA and Google Scholar for depositing copies of articles that should be available in open access.

### 1.2 Study context and objectives

We observed that many of the studies on the open access of publications have focused on universities and, to a lesser extent, on research centres, even though they are highly competitive entities with a high-impact scientific output. Our objective was to analyse research centres, with a specific focus on a network of Catalan research centres belonging to the Catalan government’s CERCA Institution that have achieved high levels of scientific output and international visibility. In doing so, our aim was to contribute fresh data to complement the existing studies, which are highly diverse in terms of both geographical scope and centre type.

The research centres belonging to the CERCA Institution are independent bodies that form part of Catalonia’s public research, development and innovation system. A total of 9,000 researchers work in the centres. Forty percent of their funds come from the Catalan government (40%), while the remainder comes from competitive calls, service contracts, the exploitation of patents and sponsorships. The CERCA centres are divided into six thematic areas: science (eight centres), life sciences (five centres), health sciences (18 centres), social sciences (two centres), engineering and architecture (five centres) and the humanities (three centres). Appendix I includes a specific list for each centre.

Director Lluís Rovira [22] reported that, considered jointly, the CERCA centres have been the eighth largest recipient of European funds since 2014, behind the National Centre for Scientific Research (France), the Max Planck Institutes (Germany) and universities, including Oxford, Cambridge and University College London. Moreover, they have a strong scientific output in relation to Catalonia’s total output, as revealed by Méndez-Vásquez [23], who presented data for the 1995–2009 period.

The overall objective of our study was to analyse the open access availability of CERCA research centre publications to determine the extent to which authors use open access journals, repositories, social networks and other websites to disseminate their research results. The specific aim of the present study was to respond to the following questions:

Q1: In what percentage and in what open access format (journals, repositories, social networks, etc.) is CERCA’s scientific output available online?
Q2: Are there differences between thematic areas when it comes to open access publications?
Q3: To what degree are CERCA’s articles published in open access journals, hybrid journals or journals with embargo periods?
Q4: How many of CERCA’s articles are archived in repositories, and which repositories are used most frequently?
Q5: How many of CERCA’s articles are available through academic social networks?

## 2 Materials and methods

### 2.1 Information sources

The SCIE, SSCI and AHCI databases of WoS (Clarivate) were used to identify the scientific output of the CERCA centres for the 2011–2015 period, and to extract basic the data concerning such output, based on the work that has been carried out by the BAC group (Bibliometrics and Evaluation of Science) of the Catalan Foundation for Research to monitor affiliations. For each research centre, a search by affiliation (OG = Organization-Enhanced) was made in the WoS database. The search included all the variants of the names of each centre.

Other potential sources were: the reports published on the websites of each centre, the annual reports issued by the CERCA Institution and the Scopus database (Elsevier). After having analysed these three sources, we concluded that it was not possible to retrieve the information required to conduct the study using them. With respect to Scopus, which has greater coverage of the social sciences, arts and humanities than WoS, no tool was available in this database to unify all the variants of the corporate name of the 40 centres analysed.

### 2.2 Definition of the samples

Out of the 43 current CERCA centres, 40 were included in the study; the Catalan Institute for Cultural Heritage Research (ICRPC) was excluded since its articles are not indexed in WoS. The Catalan Institute of Climate Sciences (IC3) and the Institute of Predictive and Personalized Medicine of Cancer (IMPPC) were also excluded since they are not currently listed as CERCA centres, even though they existed during the study period (2011–2015).

A total of 44,423 articles were published between 2011 and 2015. These were distributed very disproportionately between the 6 CERCA research areas and the 40 CERCA centres included in the study, since the number of researchers and other indicators, such as the number of projects, vary greatly between centres. We therefore opted for an approach based on 6 independent samples for each thematic area to which CERCA centres are linked, combined with an stratified random sample inside each area proportional to the output of each centre. The size of the six samples for each CERCA area was set at a confidence level of 95% and a sample error of 3%.Once the size of each sample had been established (Table 1), the articles to be included from each CERCA centre was obtained with a random extraction process but proportional to the number of articles produced by each centre within its thematic area.

**Table 1:** Articles by thematic area published by CERCA centres 2011–2015 and number of articles included in the six samples.

Centre or thematic area	Articles 2011–2015	Articles in each sample
Science	6,111	909
Life sciences	3,259	805
Medical and health sciences	33,101	1,034
Social sciences	95	88
Engineering and architecture	1,497	624
Humanities	360	270
Total	44,423	3,730

The references for the 3,730 articles were randomly extracted from Web of Science (WoS), without stratifying by year. This article selection process took place in July 2017.

For each article selected, all fields in the full record and cited references (“Full record and cited references”) were extracted from Web of Science. After the extraction process, the data corresponding to the following fields were loaded into the database:

- Title of the article
- Authors
- DOI
- Title of publication
- Year
- Volume and issue
- WoS identifier
- Research area
- Funding (FU: funding agency; FX: funding text)
- Open access (inclusion in DOAJ)

### 2.3 The “availability” concept

Our research focused on the degree of online availability of articles published by Catalonia’s CERCA research centres. The concept of open access was not applied in its most complete form, since we did not analyse the permitted uses of the copies found online (versions with Creative Commons or other licences, which allow users to reuse them), but rather their availability for free-of-charge consultation by other researchers and users in general. Thus, the study analysed the availability of online articles without the need to subscribe, purchase the article or register in any closed web environment, such as an intranet and restricted access environments. We therefore opted for the broader definition of open access proposed by Peter Suber [24], analysing the “gratis OA” of this research centres that is to say the articles in free online access (removal of price barriers) and not the “libre OA”, the articles free online access with additional re-use rights (removal of price and some/all permission barriers). That involved analysing all website types that allow users to consult an article by a CERCA centre.

This is an important issue, also highlighted by the authors of the Science-Metrix study [12] and by Martín-Martín [25].

### 2.4 Search procedure used and data collected for the study

The open availability of the articles in the samples was analysed between August and December 2017. Following the methodology used in previous studies such as Archambault (6) and Laakso (9), we searched the internet for each article using Google Scholar and Google, according to the procedure used by online researchers who do not consult specific websites such as journal portals, repositories (ArXiv, etc) or the Sci-Hub database.

In the case of Google Scholar, a maximum of 20 different copies for each article was consulted. This limit was generally sufficient for most centres, since the number of copies found for each article was lower. However, in the case of some centres in the field of science (ICFO, IFAE and IEEC), Google Scholar returned a higher number of copies for some articles, which is why it was necessary to define a limit on the number of copies. In the case of Google, the first 10 hits were consulted, which normally correspond to the number of hits in the first screen of results. Also, the search procedure was carried out using the full title of each article, without quotation marks, first on Google Scholar and then on Google, in accordance with the methodology used in comprehensive research projects such as those carried out by Archambault [6] and Pinowar [10]. Archambault reported that Google and Google Scholar presented a significant overlap, but each produced different results. The search process was systematized as much as possible. No robots or APIs were used.

Whenever an article was found on a website, the full text was accessed to verify that the content actually corresponded to the full text, and other documents with the same title, such as presentations of oral communications and abstracts of articles, were discarded.

Once the articles that were not available in open access had been discarded (those that were accessible only through subscription or in printed versions), each copy found was assigned to a unique category in accordance with the following classification:

1. Article published in open access in a digital journal:
  - The article has been published in an open access journal or in a journal aggregator such as Scielo and Redalyc.
  - The article has been published in a hybrid journal or in a journal with an embargo period.
2. Article archived in a repository:
  - Institutional repository within the CERCA centre itself.
  - Institutional repository at a Catalan university linked to the CERCA centre.
  - Institutional repositories of other organizations worldwide.
  - A cooperative or consortium, such as RECERCAT (promoted by the Consortium of University Services of Catalonia), Scientia (Ministry of Health of the Catalan government), HAL (French repository maintained by the Centre for Direct Scientific Communication) or DigitalCSIC (maintained by the Spanish National Research Council).
  - Subject-based repository such as arXiv, PubMed Central or SSRN.
  - Orphan repository such as Zenodo.
3. Other options:
  - Personal websites belonging to researchers or research groups/projects websites.
  - Academic social networks such as Academia.edu or ResearchGate.
  - Other: websites owned by companies, associations and banks, and websites such as CiteSeer, Semantic Scholar or CORE.

Within the same category of the three options analysed (journals, repositories and other options), we collected as many different copies as were found. For each copy found, information on the version type (published, accepted, author version or unknown version) was extracted from the document or the article metadata.

The journals in which open access articles were found were divided into two categories: those listed in the Directory of Open Access Journals (only those in which all articles were available in open access and published in open access immediately, without any embargo period, were considered open access journals), hybrids (open access publication is optional and based on payment of an APC) and those that publish in open access after an embargo period. Note that making a distinction between open access journals and hybrids can be a challenging exercise, as highlighted by Fathli (2014): “The amount of *hybrid* OA is difficult to measure with accuracy (and potentially very labour intensive), since there are no easily available and reliable data” (p. 2).

### 2.5 Search period

In the first quarter of 2017, a pilot test was carried out with a total of 125 articles by six CERCA centres. This test was used to validate the proposed methodology and to verify that the requirements defined for the creation of the database for collecting all the information for the study were adequate. During the second quarter, the articles to be included in the samples were selected and loaded into the database. Finally, the work to locate the 3,740 sample articles online was carried out between August and December of that same year.

Since the data were collected in 2017, articles published between 2011 and 2015 that may have been subject to an embargo period were probably already available in open access. We considered the most common embargo threshold to be 18 months.

## 3. Results and discussion

### 3.1 Output available in open access

Of the 3,730 CERCA articles analysed, 75,8% (2,828 articles) had one or more than one version available online in open access. The remaining 24,2% (902 articles) were not found in open access versions and, therefore, in principle, could be consulted only through a subscription or in their printed version. This calculation took account of all open access types included in the study, i.e. articles published in open access journals and articles with copies archived in repositories or included on any other type of website, including personal websites belonging to researchers and research groups or projects, social networks and other types of website such as Google Books and Internet Archive.

However, this required an approach with a more precise definition of the concept of open access. In the Danish Open Access Barometer, Elbaek [14] defines open access as “research literature that is published on the Internet, either in an open archive and/or in an Open Access journal, in a way that enables public access”. Similar studies on open access, like Fathli’s [13] have also advised against including personal websites, research group websites and social networks.

By applying this more accurate approach to measuring the open access availability of the output of an institution, country, etc., the degree of open access of CERCA centre output decreased considerably, since just 52% of its scientific output (1,940 articles) had one or more than one open access version available in repositories or in journals, whether pure open access or hybrid journals, or journals that publish content in open access after an embargo period. The remainder (1,790 articles) had no online versions available through the green or gold routes.

The 2011–2015 results for the CERCA centres were consistent with, and even exceeded, those produced by Archambault [6] based on an analysis of 320,000 articles published between 2004 and 2011. The results revealed how countries such as Switzerland, Brazil, the Netherlands and the United States and disciplines such as biomedicine, biology, mathematics and statistics reached a tipping point in 2011, when more than 50% of articles were available in open access through the green and gold routes.

The degree of open access of the CERCA centres scientific output was found to be in line with the results of the studies of open access publications in the Seventh Framework Programme [7], which revealed that 54.2% of the articles resulting from European projects between 2007 and 2013 were published in open access. This figure increased significantly in recent years to reach 67% in 2014 and 80% in 2015.

Based on the results obtained in the analysis of three samples of 100,000 articles, a second large-scale study by Pinowar [10], estimated that at least 28% of the academic literature was available in open access through the green and gold routes. The results for the CERCA centres far exceeded these findings.

Journals and repositories were the publishing channels most likely to guarantee the availability and integrity of open access content, since, as pointed out by Fathli [13], they provide sustainability and integrity of the files to secure their long-term availability. The author also considered the personal websites of researchers and academic social networks to be non-robust and non-sustainable in the long term. Finally, a study by Lakso [9] showed how articles on personal or institutional websites had a high chance of becoming unavailable after three years.

#### 3.1.1 Copies found

With respect to the number of copies found and having the aim to provide a more complete vision of availability, of the 3,730 articles in the samples, and more specifically, of the 2,828 articles for which at least one open access version was found, 9,868 copies were located (Table 2). Of these, 24.3% were copies located on social networks and 19.1% (1,886) were copies archived in subject-based repositories. A further 18.7% (1,842 copies) were found on other types of website and 12.9% (1,275 copies) were versions of articles in open access journals, hybrid journals or journals with an embargo period.

**Table 2:** Copies of articles found, according to type of website.

Type of website	Copies found	%
Hybrid journals or journals with embargo	650	6.,6%
Open access journals	625	6.3%
Total journals	1,275	12.9%
Repositories of linked institutions	401	4.1%
Institutional repositories of other organizations	807	8.2%
Subject-based repositories	1,886	19.1%
Cooperative repositories	181	1.8%
Orphan repositories	56	0.5%
Total repositories	3,331	33.7%
Personal, research group and research centre websites	1,027	10.4%
Academic social networks	2,393	24.3%
Other: websites of companies, associations, etc.	1,842	18.7%
Total other website types	5,262	53.4%
***Total copies found***	***9*,*868***	***100%***

Beyond the availability of an article in open access, the existence of multiple copies of the same paper is important in terms of the probability of encounter between the paper and the reader. On the other hand, the laborious work of searching for the different copies allows us to see if it complies with the most canonical forms of open access ensuring not only availability but also preservation, according to mandates.

With regard to the total number of copies found, repositories represented the most commonly used type of website to provide access to scientific documentation, followed by academic social networks. These results contrasted with those of Laakso [9] in a study on publications by Hanken School of Economics between 2012 and 2014, in which the author concluded that academic social networks had replaced research group and project websites and repositories to become the most popular means of providing access to the scientific output of researchers. Note that, while Laakso collected a maximum of three copies on the internet, our study was much more exhaustive in this regard, since we collected up to 20 copies in Google Scholar and analysed the first 10 Google hits. Nevertheless, both studies, which were based on publications practically the same period, revealed that social networks now play a key role when it comes to publishing researchers’ scientific communication in open access.

The findings for the CERCA centres were in stark contrast to those obtained in another study [20]. Based on an analysis of articles published in 2014 by researchers from 13 Spanish universities, the author found that only 11% of the articles had been made public in repositories (although the study only included the repositories of the universities analysed), while 58% of the articles had been shared on ResearchGate. These data are especially interesting, since the two studies focused on institutions in the same country, although Borrego’s study centred on universities and our study looked at research centres.

In any case, there is a clear lack of knowledge of institutional repositories among researchers and, at the same time, a strong interest in academic social networks, which are regarded as easy to use and effective in terms of disseminating research results.

#### 3.1.2 Versions of the copies found

As shown in Table 3, the publisher’s final version, i.e. the type formatted for publication by journal publishers, was found in 75.3% of cases. This percentage is high given that just 12.9% of the copies were found in open access or hybrid journals (i.e. those guaranteed to contain the publisher’s final version). It would therefore be interesting to analyse in future the extent to which researchers who have shared the remaining 62.4% online in personal websites, academic social networks or other type of webs have checked and complied with publishers’ rules regarding the use of articles. Journals’ copyright policies are outlined on their websites, in contracts signed by authors and also in the SHERPA/RoMEO database.

**Table 3:** Versions found.

Version	Number of copies	%
Publisher’s final version	7,429	75.3%
Accepted version	254	2.6%
Author’s version	580	5.8%
Unknown version	1,605	16.3%
Total	9,868	100%

#### 3.1.3 Single copies found

For 749 articles in the samples (20% of the total), only one copy was found online. Table 4 shows their distribution by website type and also the percentage compared to the total number of copies. The most striking finding was that almost half of these single copies (369 articles) were found on academic social networks, which reveals the popularity of these channels among authors. Repositories, the main competition for social networks in terms of attracting articles published in subscription journals, represented just 20.1% of the total (all types were included). This huge gap in the percentages can be explained by the ease of use and features presented by social networks with respect to repositories, but it is important to bear in mind that academic social networks offer no control over copyright compliance, unlike repositories.

**Table 4:** Articles found on one site only.

Category	Total copies found	%	Articles with one single copy	%
Academic social networks	2,393	24.3%	369	49.3%
Hybrid journals or journals with embargo	650	6.6%	98	13.1%
Personal, research group and research centre websites	1,027	10.4%	60	8%
Other: websites of companies, associations, etc.	1,842	18.7%	45	6%
Subject-based repository	1,886	19.1%	42	5.6%
Repository of a linked university	401	4.1%	34	4.5%
Cooperative repository	181	1.8%	30	4%
Institutional repository of another organization	807	8.2%	28	3.7%
OA journal	625	6.3%	26	3.5%
Orphan repository	56	0.5%	17	2.3%
Total	9,868	100	749	100%

Other studies like Laakso’s [9] have obtained similar findings: academic social networks represent the type of website that contain most single articles that have been published in subscription journals.

### 3.2. Open access by thematic area

Table 5 shows the percentage of articles found that were available in open access, broken down by thematic area. This comparison based on relative values is merely indicative, since the samples vary in size. These data reveal that, for all thematic areas addressed by CERCA centres, the scientific output available in open access was high, given that it exceeded 70% in all six areas and reached as much as 89.8% in the case of social sciences, although this information must be considered in the context of the low output levels of these centres in WoS.

**Table 5:** Open and closed articles according to thematic area.

Thematic area	Open access	Closed access	Total	% Open access
Science	744	165	909	81.8%
Life sciences	570	235	805	70.8%
Medical and health sciences	756	278	1,034	73.1%
Social sciences	79	9	88	89.8%
Engineering and architecture	472	152	624	75.6%
Humanities	207	63	270	76.7%

A recent study by Science-Metrix [12] used an approach based on five thematic areas (health sciences, natural sciences, applied sciences, economics and social sciences, and arts and humanities). Some were similar to the CERCA areas. All except Science and Engineering and Architecture have an equivalent. It was therefore considered useful to compare the two studies even if they were not equivalent: Science-Metrix study was not based on a sample but on data for the entire population (i.e. all articles indexed on Web of Science and Scopus during the 2006–2015 period). Table 6 shows the data from the two studies without the CERCA centre thematic area “sciences” and “engineering and architecture”, since those were not differentiated by Science-Metrix.

**Table 6:** Comparison of the percentages of open access for CERCA and Science-Metrix according to thematic area.

Thematic area	% OA CERCA	% OA Science-Metrix
Life sciences / Natural sciences	71	55
Medical and health sciences / Health sciences	73	59
Social sciences / Economics and social sciences	90	44
Humanities / Arts and humanities	77	24

First, it is clear that there is a greater difference between the percentages of the thematic areas in the Science-Metrix study, which ranged from 59% for health sciences to 24% for arts and the humanities. More consistency can be observed in the case of the CERCA centres, since the percentages ranged only from 71% to 90%. The second observation is that life sciences (or natural sciences) and medical sciences (or health sciences) were the two areas in which the two studies differed least. Third, the wide disparities in the areas of economics and social sciences and arts and humanities should be treated with caution, since they can be explained by the effects of pure chance, given that the number of CERCA articles in these areas was very low.

Our results can also be compared to those of a study by Björk [3] on a sample of 1,837 articles in different subject areas. The author found considerable differences in terms of open access availability according to disciplines, since the results ranged from 13% in chemistry to 33% in earth sciences. The percentages were similar to those of the CERCA centres.

Finally, we present a comparison of the different areas based on the main website categories analysed. Table 7 reveals that, in all cases, the generic category “Other” is the most commonly used type of website for publishing articles online, particularly with respect to engineering.

**Table 7:** Locations of the documents found, according to thematic area.

	Sciences	Life sciences	Medical and health sciences	Social sciences	Engineering and architecture	Humanities
OA journals	12.3%	18%	26.5%	8.9%	7.8%	13.7%
Repositories	41%	27.7%	26%	33.2%	20.9%	20%
Other	46.7%	54.3%	47.5%	57.8%	71.3%	66.3%

### 3.3 Articles published in journals

As shown in Table 8, 84.8% of the articles found were published in subscription journals (including hybrid journals and those that publish the content after an embargo period), a very high percentage with respect to open access journals. These data were obtained by searching DOAJ for the journal titles in which articles were found in open access format.

**Table 8:** Articles published in subscription journals versus open access journals.

	Articles	%
Articles published in open access in subscription journals	624	19.7
Articles published in closed access in subscription journals	2,539	80.3
Articles in subscription journals—Total	3,163	84.8
Articles in open access journals	567	15.2
Total articles	3,730	100

When the 3,163 articles published in subscription journals were analysed, 19.7% (624 articles) were found to be published openly, either because the authors had paid APCs for their immediate open publication or because the journal has an embargo period after which articles become openly available online.

### 3.4 Articles archived in repositories

Only 40.4% of the articles in the samples (1,509 articles) were found in repositories of any type (institutional, subject-based, etc.). This total (Table 9) was divided almost equally between open articles in open access journals, hybrid journals and journals with an embargo period (755 articles), and closed articles in subscription journals (754 articles).

**Table 9:** Articles in repositories.

	Articles	%
Articles not found in repositories	2,221	59.6%
Articles found in repositories and published in open access or hybrid journals	755	20.2%
Articles found in repositories and published in closed access in subscription journals	754	20.2%
Total	3,730	100%

The study published by the Research Information Network [15], on the open access situation in the United Kingdom found that just 15% of the articles published in subscription journals in 2014 were archived in institutional or subject-based repositories, a result that is highly consistent with the results for the CERCA centres (20.2%).

When the repositories containing the 3,730 articles in the samples were analysed in greater detail, an interesting fact emerged: no copies were found in the institutional repositories of CERCA centres. This is somewhat predictable, since no clear policy is currently in place regarding the CERCA network or specific centres. Second, of the 2,820 articles for which at least one copy was found online, 400 (14%) were found in at least one institutional repository at Catalan universities with links to the CERCA centres due to the centres’ location within or near the universities. This finding is underpinned by the fact that agreements are in place regarding the centres’ use of these repositories, either because the CERCA authors of these publications have two affiliations (i.e. they are linked to both a research centre and a university), or because one of the co-authors is a member of one of these universities.

Finally, an analysis of the geographical distribution of the repositories used by the authors of the articles in the samples (Table 10) revealed that a third of the copies are located in repositories in Catalonia and a further third in repositories in Europe. This table does not include copies in subject-based repositories, because these cannot be assigned to a country. The total number of copies is therefore lower than the 1,509 in Table 9.

**Table 10:** Articles archived in repositories according to the geographical area of the repository.

Geographical area	Articles	%
Catalonia	413	34.1%
Rest of Spain	216	17.8%
Rest of Europe	401	33.1%
North America (Canada and the US)	111	9.2%
South America	38	3.1%
Oceania	21	1.8%
Africa	3	0.2%
Asia	8	0.7%
Total	1,211	100%

### 3.5 Articles on academic social networks

A little over half of the articles in the samples (1,881) had a copy on academic social networks (Table 11). This is a little higher than the figure obtained by Laakso [9] in a study on the output of Hanken School of Economics (HSE), which found that the availability of articles by this institution on social networks was 41% in 2012, 49% in 2013 and 37% in 2014. The time interval between 2013–2014 and 2017, when we performed our analysis, goes some way to explaining this difference, since the use of academic social networks has increased in recent years. However, it should be noted that most of the articles found in these academic social networks (29.5%) were published in closed access in subscription journals.

**Table 11:** Articles on academic social networks.

	Articles	%
Articles posted on social networks and published in open access	789	21%
Articles posted on social networks and published in closed access in journals	1,092	29.5%
Articles not posted on academic social networks	1,849	49.5%
Total	3,730	100%

The two most commonly used networks were ResearchGate and Academia, while networks such as ScienceOpen and Scipedia made only a token appearance, as shown in Table 12. In this case, the data were obtained based on the number of copies found.

**Table 12:** Use of academic networks (based on copies found).

	Copies	%
ResearchGate	1,543	63.4
Academia	880	36.2
ScienceOpen	8	0.3
Scipedia	2	0.1

Copies of pirated articles uploaded to websites like Sci-Hub and those that are open on social networks, this is what Björk [26] calls the black Open Access. The author also considers that ResearchGate is the main academic social network used by researchers.

An analysis of usage by thematic area (Table 13) revealed that ResearchGate was the most commonly used network by researchers at the CERCA centres (or by the co-authors with whom they publish) across all disciplines, with the exception of humanities (in which Academia was by far the most popular network) and the social sciences (in which the number of copies was the same).

**Table 13:** Use of ResearchGate and Academia according to thematic area (copies found).

Thematic area	ResearchGate	%	Academia.edu	%
Sciences	467	30.3	168	19.1
Life sciences	364	23.6	178	20.2
Medical and health sciences	387	25.1	247	28.1
Social sciences	13	0.8	13	1.5
Engineering and architecture	212	13.7	128	14.5
Humanities	100	6.5	146	16.6
Total	1,543	100	880	100

### 3.6 Degree of compliance with the Spanish Science Act

Article 37 of the Spanish Science Act [27] stipulates that articles resulting from projects partially or fully financed with public funds must be disseminated in open access repositories. According to our data, copies of just 37.9% of the articles (680) were found in a repository, which would indicate that there is little compliance with this law among the CERCA centres. The results (Table 14) were obtained using the fields in Web of Science to retrieve information on an article’s means of funding (FX and FU). We then selected the articles that were funded by state projects and published between 2013 (when this law came into force) and 2015, and checked whether they were archived in repositories.

**Table 14:** Availability of articles resulting from state projects in repositories (2013–2015).

	Articles	%
Archived in repositories	680	37.9
Not archived in repositories	1.114	62.1

Borrego’s study [20], which was conducted two and a half years after the implementation of the law, showed that 58.4% of the articles deriving from projects financed by the Spanish government had at least one copy available in open access one year after publication. However, 23.8% of these were published via the gold route, 21.8% were published via the green route and 12.8% were found on websites or on academic social networks. When their data on repositories (21.8%) was compared to ours (37.9%), the difference was not as large as it first seemed when their overall result was considered (58.4%).

**Fig 1:**
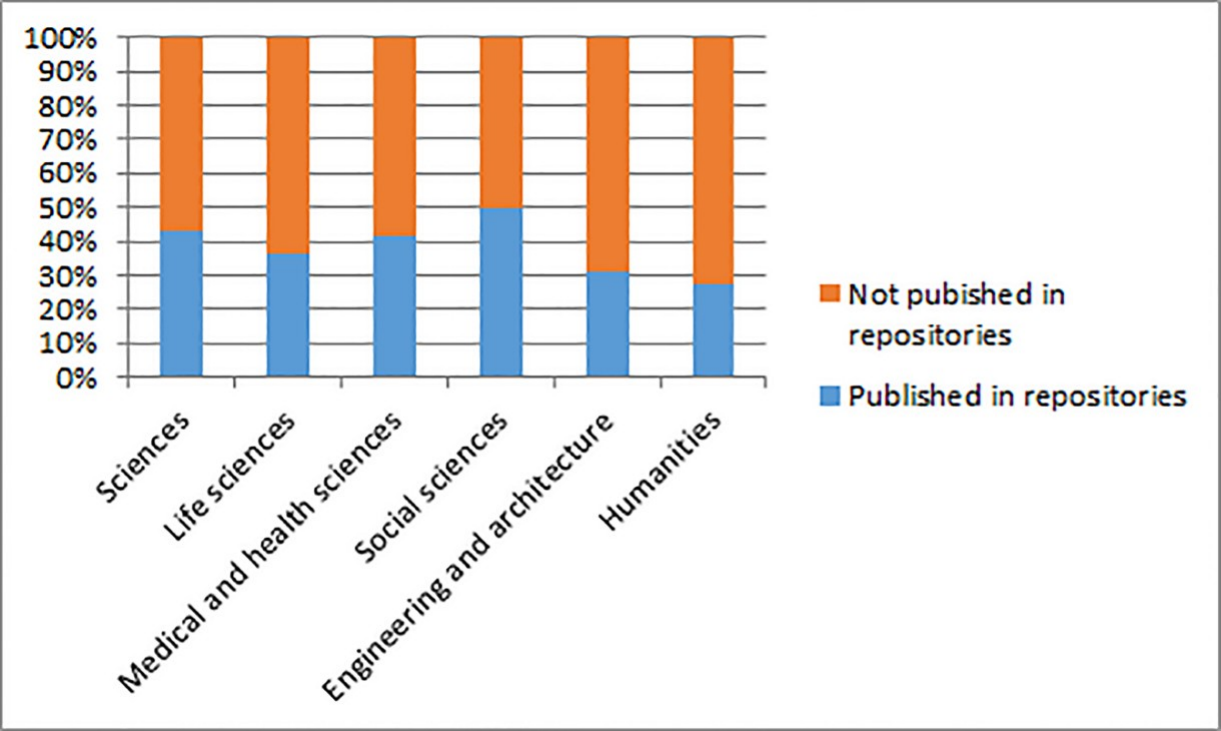
Degree of compliance with the Science Act by thematic area.

When the distribution by CERCA thematic area (Table 15) was analysed, the fields of sciences and the medical and health sciences presented the highest percentages (Fig 1). It is important not to attach too much weight to the 50% achieved by social sciences, because the number of articles analysed was low.

**Table 15:** 2013–2015 articles linked to competitive state projects published in repositories.

CERCA thematic area	Articles in repositories	%	Articles not published in repositories	%
Sciences	198	42.9	264	57.1
Life sciences	148	36.5	257	63.5
Medical and health sciences	193	41.9	268	58.1
Social sciences	7	50	7	50
Engineering and architecture	90	30.9	201	69.1
Humanities	44	27.3	117	72.7

## 4 Conclusions

The CERCA centre network provides Catalonia with powerful research infrastructure, as it has achieved high levels of scientific output and attracts a great deal of research funding.

Based on a sample of 3,730 articles published by CERCA’s researchers between 2011 and 2015 our findings indicated that 75.8% of the articles were available online in open access format, applying the broadest definition of open access. Regarding the articles found in open access according to thematic areas, all six areas (science, life sciences, medical and health sciences, engineering and architecture and humanities) exceeded 70% of articles in open access. When only articles in repositories or open access journals were taken into account, just 52% of the scientific output was openly available.

The study also revealed that the number of articles published in open access journals (567) was very similar to the number of articles published in hybrid magazines or journals with embargo periods (624). Another finding is that researchers at the CERCA centres continued to publish mainly in subscription journals and/or did not pay APCs to publish them in open access.

With respect to repository usage, the study found that just 40.4% of the articles in the samples were archived in a repository, being the subject repositories the heaviest used. Finally, 50% of the articles were posted in academic social networks, the most popular of which was ResearchGate.

It is important to bear in mind that our data merely provide a snapshot of the situation. As highlighted by Lakso [9] a few years ago, many articles on personal or institutional pages are highly likely to disappear after a certain period of time. Regarding academic social networks, Jamali [28] recently calculated that half of the articles they contain are posted illegally, a fact that led some publishers to threaten these social networks with legal action at the end of 2017 if they did not remove all content posted in violation of the law [29]. Thus, if we decided to repeat our study in the future, the results are likely to be quite different.

## Appendix I: Research centres by thematic area

Sciences:

- Centre de Recerca Matemàtica (CRM)
- Institut Català d'Investigació Química (ICIQ)
- Institut Català de Nanociència i Nanotecnologia (ICN2)
- Institut Català de Paleontologia Miquel Crusafont (ICP)
- Institut Català de Recerca de l'Aigua (ICRA)
- Institut d'Estudis Espacials de Catalunya (IEEC)
- Institut de Ciències Fotòniques (ICFO)
- Institut de Física d'Altes Energies (IFAE)

Life sciences:

- Centre Tecnològic Forestal de Catalunya (CTFC)
- Centre de Recerca Ecològica i Aplicacions Forestals (CREAF)
- Centre de Recerca en Agrigenòmica (CRAG)
- Centre de Recerca en Agrotecnologia (Agrotecnio)
- Institut de Recerca i Tecnologia Agroalimentàries (IRTA)

Medical and health sciences:

- Centre de Medicina Regenerativa de Barcelona (CMR[B])
- Centre de Regulació Genòmica (CRG)
- Institut Català de Ciències Cardiovasculars (ICCC)
- Institut Hospital del Mar d'Investigacions Mèdiques (IMIM)
- Institut d'Investigacions Biomèdiques A. Pi i Sunyer (IDIBAPS)
- Institut d'Investigació Biomèdica de Bellvitge (IDIBELL)
- Institut d'Investigació Biomèdica de Girona (IDIBGI)
- Institut d'Investigació Sanitària Pere Virgili (IISPV)
- Institut d'Investigació en Ciències de la Salut Germans Trias i Pujol (IGTP)
- Institut de Bioenginyeria de Catalunya (IBEC)
- Institut de Recerca Biomèdica (IRB Barcelona)
- Institut de Recerca Biomèdica de Lleida (IRB Lleida)
- Institut de Recerca Contra la Leucèmia Josep Carreras (IRCL)
- Institut d'Investigació Biomèdica Sant Pau (IIB Sant Pau)
- Institut de Recerca de la Sida (IrsiCaixa)
- Institut de Salut Global de Barcelona (IS Global)
- Vall d'Hebron Institut d'Oncologia (VHIO)
- Vall d’Hebron Institut de Recerca (VHIR)

Social sciences:

- Centre d'Estudis Demogràfics (CED)
- Centre de Recerca en Economia Internacional (CREI)

Engineering and architecture

- Centre Internacional de Mètodes Numèrics en Enginyeria (CIMNE)
- Centre Tecnològic de Telecomunicacions de Catalunya (CTTC)
- Centre de Visió per Computador (CVC)
- Institut de Recerca en Energia de Catalunya (IREC)
- Internet i Innovació Digital a Catalunya (I2CAT)

Humanities:

- Institut Català d'Arqueologia Clàssica (ICAC)
- Institut Català de Recerca en Patrimoni Cultural (ICRPC)
- Institut Català de Paleoecologia Humana i Evolució Social (IPHES)
